# Frontolimbic alpha activity tracks intentional rest BCI control improvement through mindfulness meditation

**DOI:** 10.1038/s41598-021-86215-0

**Published:** 2021-03-25

**Authors:** Haiteng Jiang, James Stieger, Mary Jo Kreitzer, Stephen Engel, Bin He

**Affiliations:** 1 Department of Biomedical Engineering, Carnegie Mellon University, Pittsburgh, PA USA; 2 University of Minnesota, Minneapolis, MN USA

**Keywords:** Brain-machine interface, Cognitive neuroscience

## Abstract

Brain–computer interfaces (BCIs) are capable of translating human intentions into signals controlling an external device to assist patients with severe neuromuscular disorders. Prior work has demonstrated that participants with mindfulness meditation experience evince improved BCI performance, but the underlying neural mechanisms remain unclear. Here, we conducted a large-scale longitudinal intervention study by training participants in mindfulness-based stress reduction (MBSR; a standardized mind–body awareness training intervention), and investigated whether and how short-term MBSR affected sensorimotor rhythm (SMR)-based BCI performance. We hypothesize that MBSR training improves BCI performance by reducing mind wandering and enhancing self-awareness during the intentional rest BCI control, which would mainly be reflected by modulations of default-mode network and limbic network activity. We found that MBSR training significantly improved BCI performance compared to controls and these behavioral enhancements were accompanied by increased frontolimbic alpha activity (9–15 Hz) and decreased alpha connectivity among limbic network, frontoparietal network, and default-mode network. Furthermore, the modulations of frontolimbic alpha activity were positively correlated with the duration of meditation experience and the extent of BCI performance improvement. Overall, these data suggest that mindfulness allows participant to reach a state where they can modulate frontolimbic alpha power and improve BCI performance for SMR-based BCI control.

## Introduction

A brain–computer interface (BCI) is a system that uses brain signals to control a computer or external device through bypassing neuromuscular pathways in order to reestablish or facilitate communication and agency to individuals^1–4^. One popular BCI system has been developed using noninvasive electroencephalography (EEG) to decode the user’s intention based on the sensorimotor rhythm (SMR)^1,5^. Accordingly, the performance of SMR-based BCI depends on the quality of the EEG signals, which are significantly affected by mental states^6^.

While the intuitive nature and continuous control of SMR-based BCI provides many benefits, large scale studies of SMR-based BCI control have found about 20% of participants are unable to use typical BCIs proficiently even after extensive training^7,8^. Interestingly, preliminary work has shown that participants with prior mind–body awareness training (MBAT; e.g. yoga, meditation, etc.) experience demonstrated better BCI performance compared to those with little or no MBAT experience^9,10^. Teaching users better control over their neural activity through MBAT may represent a promising complementary approach to the machine learning improvement of BCI control as BCI use is a skill that user and system acquire together^3,11^.

MBAT has become increasingly popular in recent years due to its potential physical and mental health benefits, however how meditation leads to these benefits remain unclear^12^. It has been suggested that MBAT constitutes a family of self-regulation processes that include enhanced attention control, improved emotion regulation, and altered self-awareness^13^. Additionally, neuroimaging studies have demonstrated MBAT can induce large-scale brain network reconfigurations^13–15^, particularly in the default-mode network (DMN), frontoparietal network (FPN) and limbic network (LN). Coincidentally, key brain networks that are involved in different aspects of BCI learning and neurofeedback largely overlap with meditation-related networks^16,17^. Although studies suggested meditation facilitates BCI learning, the neural mechanism of how meditation and BCI learning interact is unknown.

To address these questions, we conducted a large-scale, longitudinal study to test whether and how short-term MBAT affected SMR-based BCI learning at both the behavioral and neural levels. Specifically, we focused on the voluntarily rest BCI condition in which participants were instructed to clear their minds—where the altered self-awareness involved in MBAT would be expected to have the greatest impact on cognitive processes such as mind-wandering^18^. Mind wandering is often associated with activation of the DMN^19–21^, while reductions in mind wandering and DMN activity are consistently reported during meditation^22–24^. Altered self-awareness as mediated in part by the deep limbic brain regions was the main outcome of meditation^13,14^ and appeared to play a critical role in BCI control as well^17^. Accordingly, we hypothesized that, during the intentional resting task of BCI control, MBAT participants improved their performance by reducing mind wandering and enhancing self-awareness, which in turn would be reflected by modulations of DMN and LN activity.

## Results

### Behavior outcomes

**Figure 1:**
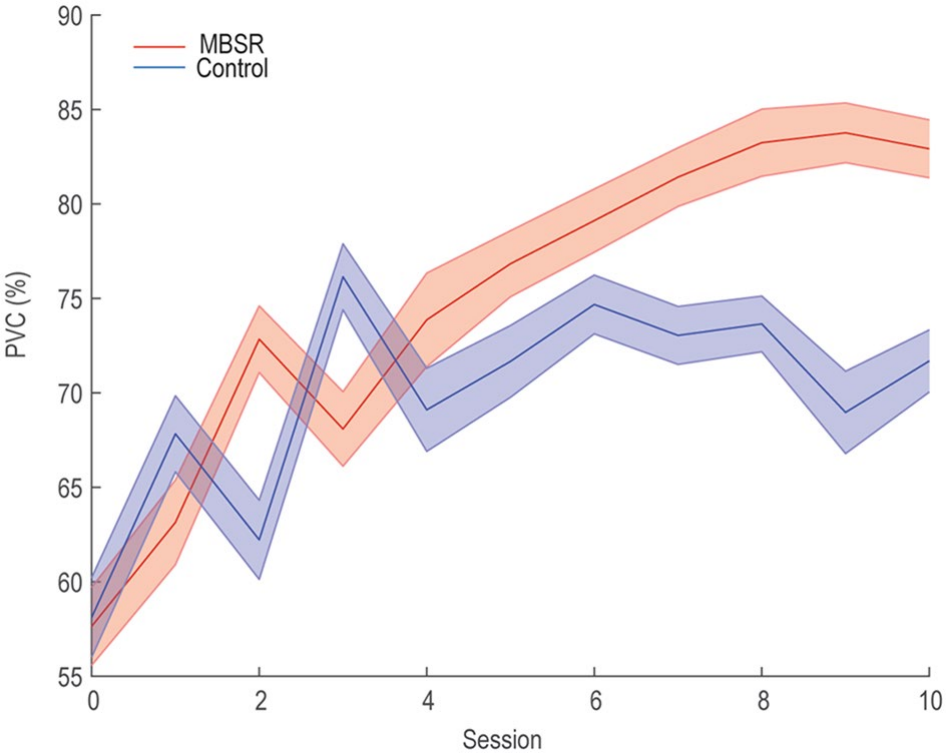
BCI performance changes over training sessions. The performance was assessed by percent valid correct (PVC).

BCI performance was quantified by a percent valid correct (PVC) metric^25^, calculated as the number of hits divided by the total number of non-timeout trials (a timeout occurred when the cursor did not contact a target within 6 s). To conduct the statistical comparisons, we used the linear mixed-effects models with fixed effects of session (levels: 11), group (levels: MBSR, control). As reported previously^18^, for the PVC, MBSR participants had greater improvements in BCI performance than controls (Fig. 1). There were significant effects in group (*F*(1,660) = 10.2, *p* < 0.005), session (*F*(10,660) = 6.16, *p* < 0.001) and the interaction between group and session (*F*(10,660) = 2.28, *p* < 0.05).

### Increased DMN and limbic alpha power was found in the MBSR group after BCI training

**Figure 2:**
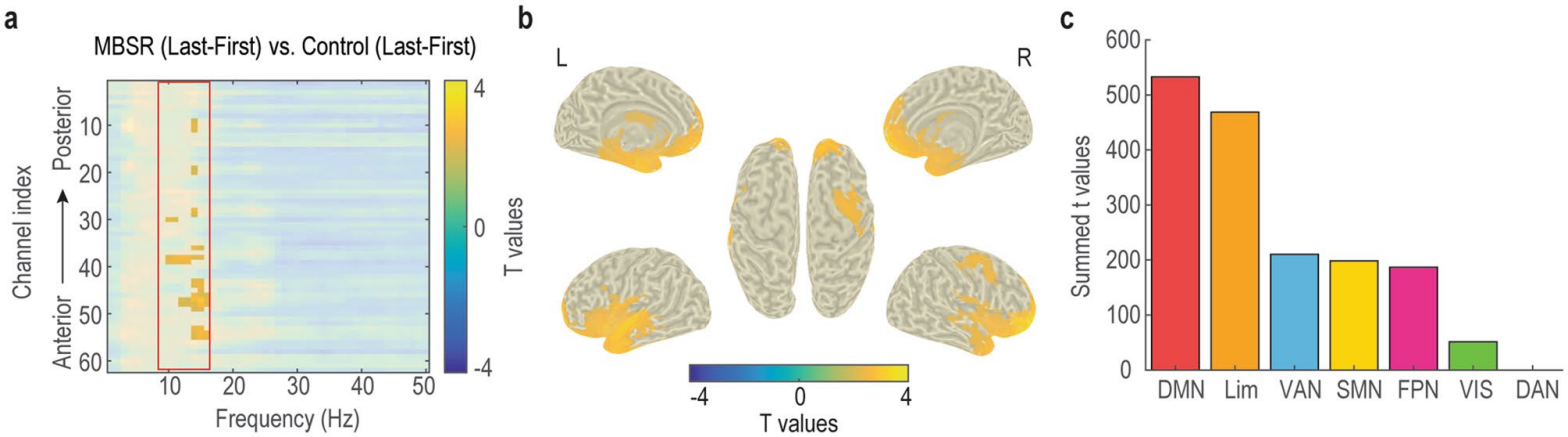
Meditation and learning interaction effects in power. (**a**) Spectral-spatial significance map identified by cluster permutation test. Only the significant elements were highlighted. (**b**) Source localization of the alpha (9–15 Hz) effect. (**c**) Summed significant t values of b in seven networks.

Next, we examined the interaction between meditation and BCI learning effects in power. The nonparametric cluster permutation test revealed a spatial-spectral cluster with significantly different activity between MBSR and control groups after BCI training (Fig. 2a). Interestingly, the significant cluster was spectrally constrained to the 9–15 Hz alpha band. A control analysis suggested that the significantly increased alpha power in MBSR was not due to pre-training and post-training resting-state alpha power differences (Fig. S1). Source analysis localized the alpha activity mainly to the frontal and limbic regions, including bilateral middle frontal gyrus, bilateral superior frontal gyrus (medial orbital), bilateral amygdala, and bilateral thalamus (Fig. 2b). When parcellated into 7 standard networks (DMN, DAN, FPN, VAN, SMN, VIS and LIM) from the literature^26^ and averaged, the most involved networks were DMN and LIM (Fig. 2c).

### Decreased frontolimbic alpha connectivity in the MBSR group after BCI training

**Figure 3:**
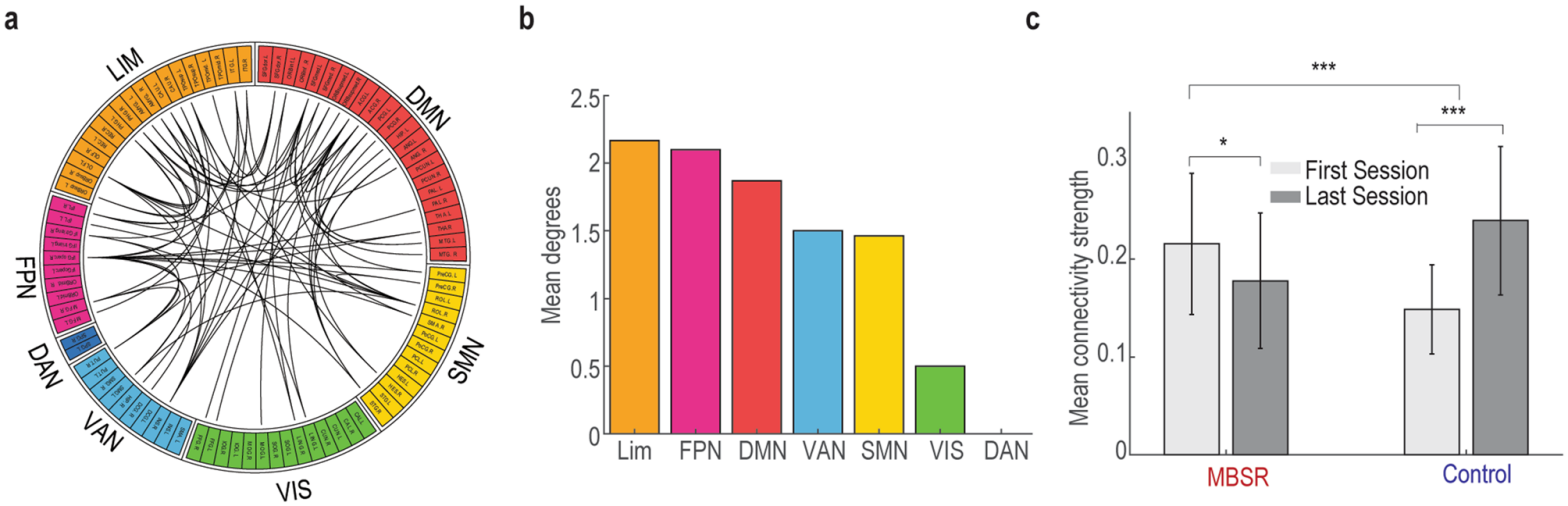
Meditation and learning interaction effects in the alpha band connectivity. (**a**) Significantly different network topographies identified by network based statistics. Each link represents a connection between two brain regions. (**b**) Network summary of (**a**) by parceling the connections into seven well-characterized resting-state networks. The mean degree is the averaged number of links within the seven networks. (**c**) Changes of mean connectivity strength within the identified network in (**a**).

After identifying the interaction effect in the alpha power, we then investigated the potential difference in functional connectivity between groups. The functional connectivity at the source level was estimated by power envelope correlation following the DICS source reconstruction^27^. When comparing the BCI learning differences (Last session vs. First session) between MBSR and control groups, there were widespread learning-related changes of functional connectivity identified by network based statistics (Fig. 3a). This network consisted of 51 regions and 72 connections, mainly involving the right inferior frontal gyrus (opercular part), left anterior cingulate and paracingulate gyri, right rolandic operculum, left superior frontal gyrus (medial part), left olfactory cortex and right insula. When parceling the connections into seven well-characterized resting-state networks, the top 3 most engaged networks were LN, FPN and DMN (Fig. 3b). Interestingly, the network responses of the MBSR and control groups were in the opposite direction: decreased network connectivity was found in the MBSR group, whereas increased network connectivity was found in the control group after BCI training (Fig. 3c).

### Correlation with meditation experience and BCI performance improvement

**Figure 4:**
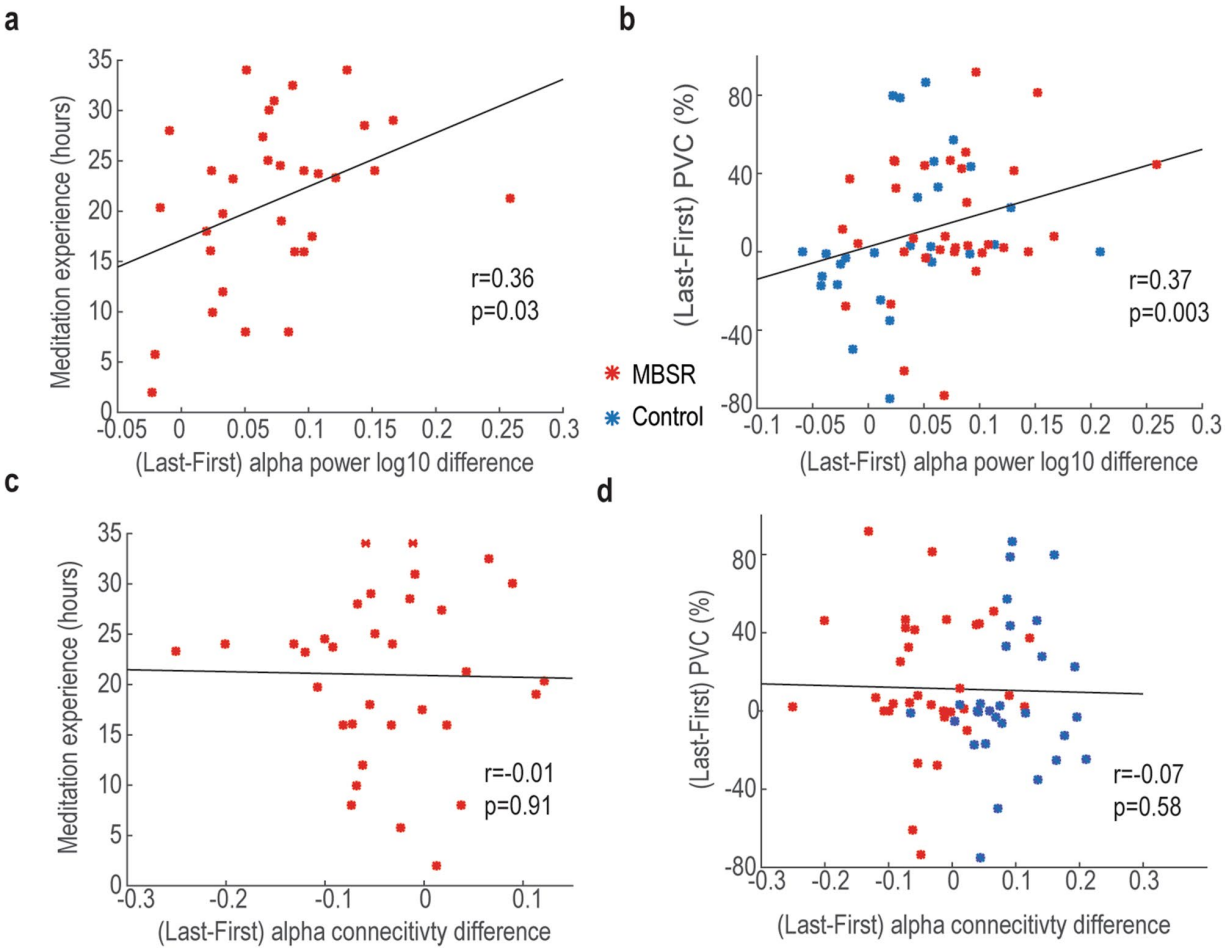
Correlation between neural signals and behavior. (**a**) Correlation between alpha power changes and meditation experience in the MBSR group. (**b**) Correlation between alpha power and PVC changes. (**c**) Similar to (**a**), but correlated with alpha connectivity. (**d**) Similar to (**b**) but correlated with alpha connectivity.

Finally, we asked whether the identified interaction effect in power and functional connectivity had behavior relevance by checking their correlations with meditation experience and BCI performance improvement (Fig. 4). That is, whether the modulations of the interaction effect in power and functional connectivity correspond to both meditation experience and BCI performance changes. To conduct the correlation analysis, we extracted the interacting power regions (Fig. 2b) and functional connectivity networks (Fig. 3a) and computed their differences between first and last BCI training sessions. Importantly, we found that the frontolimbic alpha power modulations significantly correlated with both meditation experience (*r* = 0.36, *p* = 0.03) and BCI performance changes (*r* = 0.37, *p* = 0.003) (Fig. 4a,b). However, no such effects were found in the functional connectivity analysis with either meditation experience or BCI performance changes (Fig. 4c,d).

## Discussion

Our work demonstrates a short-term MBAT intervention—mindfulness-based stress reduction (MBSR)—improved intentional rest BCI control performance by increasing DMN and LN alpha power and decreased alpha connectivity between DMN, FPN, and LN. Furthermore, modulations of DMN and LN alpha power were correlated with both meditation experience and BCI performance improvement. Overall, these findings suggest the critical roles of DMN and LN in the interaction between meditation and BCI learning and may have implications for future SMR-based BCI design and BCI training protocols.

We sought to investigate whether and how MBAT could impact BCI training. Our data showed that both MBSR and control groups had a BCI learning effect. However, the degree of improvement was significantly greater in the MBSR group compared to control group and there was a significant interaction effect between meditation and BCI learning; the BCI performance improvement in MBSR could be explained by increased DMN and LN alpha power.

Although we initially did not have a specific hypothesis about the frequency band and used a data-driven cluster permutation test to identify it, the identified band was confined to 9–15 Hz alpha power (Fig. 2a). It should be pointed out that the 9–15 Hz alpha defined here slightly deviates from the classical alpha range between 8 and 13 Hz^28^. Increased alpha power during rest or between rest and meditation have been widely reported when comparing meditation practitioners to controls^29,30^. As such, the enhanced alpha during the intentional rest BCI control could be confounded by the resting-state baseline differences after MBSR intervention. However, there were no significant resting-state alpha power differences between the MBSR and control groups after the intervention Fig. S1). Moreover, the main meditation effect (MBSR_(Last+First)_ vs. Control_(Last+First),_ Fig. S2) and BCI learning effect (Last_(MBSR+Control)_ vs. First_(MBSR+Control),_ Fig. S3) were identified other than the interaction effect. When pooling the first and last BCI sessions together and comparing between the MBSR and control groups (meditation effect), 12–15 Hz oscillatory activity over DMN and FPN was found to be increased in the MBSR group (Figure S2). When combing the MBSR and control groups and comparing the last and first BCI session (learning effect), there were widespread 4–30 Hz oscillatory activity increases after BCI training (Figure S3). Overall, these results suggest that the MBSR intervention induced frequency-specific and network-specific oscillatory power modulations in meditation, BCI learning, and their interaction.

Although the up-regulation of alpha power appears to be responsible for the gains in performance observed in our study, the nature of alpha oscillations in the interaction between meditation and BCI learning requires further study. Increased alpha activities are often believed to signify greater inhibition or gating of task-irrelevant information^31,32^, which is thought to underlie the behavior gains during working memory and sustained attention tasks^33–35^. The DMN was the most prominent network at rest and often found during the periods of mind wandering, while reductions in mind-wandering and DMN activity were consistently reported during meditation^19,22^. Therefore, the increased DMN alpha activity in MBSR participants may suggest that the MBSR group may utilize meditation skills during intentional BCI rest control and therefore reduce mind wandering by disengaging the DMN.

Moreover, increased LN alpha activity was found in the MBSR group as well, which may be associated with diminished self- referential processing and enhanced body awareness, leading to improved BCI control^13,17^. Most critically, the extent of DMN and LN alpha modulations were related to both the amount of time invested in meditation practice, and BCI performance improvement (Fig. 4a,b), indicating the neural and behavior gains can be trained in a dose-dependent manner. Additionally, these results also suggest that the default mode and limbic networks are the common networks underlying the meditation and BCI learning interaction.

Brain regions are not isolated one from another, and they connect to each other. Following the alpha power investigation, we examined the potential connectivity alterations after the MBSR intervention. A significant meditation and BCI interaction effect was found in the connectivity among the DMN, FPN, and LNs. Interestingly, the changes in alpha connectivity of the MBSR and control groups were in the opposite direction after BCI training (decreased in MBSR while increased in controls). The decreased alpha connectivity in the MBSR group may suggest their ability to limit the processing of unnecessary information during the intentional rest BCI control task^36^. However, the modulations of alpha connectivity were not associated with meditation experience or BCI performance changes (Fig. 4c,d).

A few limitations should be noted. First, we only focused on the intentional rest BCI control but not the overall task. While MBSR participants showed greater improvement in other BCI tasks, the differences were not significant compared to controls. These may question the capability of MBAT for improving other aspects of BCI control. Longer or more intensive interventions may be more effective^10,37^. Alternatively, changing the BCI task to one more akin to the skills learned through meditation may help realize greater gains in performance. Second, due to the different number of training sessions across participants, we subtracted the last and first session to represent the learning effect. It would be more optimal to apply the linear mixture model to track the learning curve with the same number of sessions in the future. Lastly, the study lacked an active control group to eliminate the possibility of expectancy effects. However, one prior study showed significantly higher BCI accuracy in the mindfulness meditation training group compared to the music training group, indicating the effects of meditation above and beyond expectancy effects^9^. Moreover, the correlation between alpha power modulations and meditation experience suggested that these observed effects were specific to mindfulness meditation practice and not due to expectation. Nevertheless, an actively controlled double-blind trial will be needed to fully establish the translational promise of our results.

In conclusion, we found that mindfulness meditation could improve the intentional rest BCI control performance, and further that frontolimbic alpha power modulations are the key neural mechanisms underlying the interaction between meditation and BCI learning. Our findings suggested that mindfulness training allows participants to enter states that modulate these signals from frontolimbic regions, and so being beneficial for SMR-based BCI control.

## Methods

### Participants and task protocol

The data analyzed here were collected from our previous large-scale longitudinal meditation BCI study approved by the Institutional Review Boards of the University of Minnesota and Carnegie Mellon University^18^. All participants provided written informed consent. Briefly, 144 participants were recruited in the intervention study and 76 of them completed all experimental requirements, which were performed in accordance with Declaration of Helsinki. Following an initial BCI performance assessment, participants were randomly assigned to either the MBSR group or the waitlist control group. The MBSR intervention aims to teach mindfulness, which is defined as “awareness that arises through paying attention, on purpose, in the present moment, non-judgmentally”^38^. After 8 weeks of the MBSR intervention or comparable waiting period, participants completed 6 or 10 BCI training sessions. The BCI task involved left (right) hand motor imagery to move a virtual cursor to the left (right), motor imagery of both hands to move the cursor up, and a voluntary rest to move the cursor down. The BCI control signal was extracted by using autoregressive (AR) spectral amplitudes of the small Laplacian filtered electrodes C3 and C4 in a 3 Hz bin surrounding 12 Hz based on the principle of maximum entropy spectral estimation^39^. Maximum entropy spectral estimation is a spectral density estimation method by choosing the spectrum that corresponds to the most random or the most unpredictable time series whose autocorrelation function agrees with the known values. Mathematically, the maximum entropy method is equivalent to least-squares fitting the available time-series data to an AR model. The duality between the maximum entropy method of spectral analysis and the AR representation of the data allows AR to get the improved spectral decomposition. After the spectral estimation, the spectral amplitudes were normalized to zero mean and unit variance. The magnitude of the cursor movement was determined by the normalized AR amplitude difference, updated every 40 ms. In this following-up study, 62 participants were included in the final analysis and we focus on the down condition. For the down BCI cursor control, participants were instructed to rest voluntarily; in other words, clear their minds, which is most similar to the meditation process. Moreover, due to different numbers of BCI training sessions across participants, we used the first and the last session EEG data to model the BCI learning effect.

### EEG acquisition

Participants were seated facing a computer monitor while wearing a 64-channel EEG cap, which was set up according to the international 10–10 system. EEG was acquired using SynAmps RT amplifiers and Neuroscan acquisition software (Compumedics Neuroscan, VA). The EEG signals were sampled at 1000 Hz and 0.1 to 200 Hz bandpass filtered with an additional 60 Hz notch filter and stored for offline analysis.

### EEG preprocessing and spectral analysis

Offline EEG data analysis was conducted using EEGLAB and Fieldtrip toolbox^40,41^. Most standard preprocessing was performed in EEGLAB (version 14.1.2). Initially, the data were bandpass filtered between 1 and 100 Hz, followed by down sampling from 1 kHz to 250 Hz. Noisy channels, identified through visual inspection, were replaced by local weighted averages interpolated through spherical splines^42^. The data were re-referenced to a common average. Ocular artifacts were removed using independent component analysis (ICA) and a template matching procedure. Briefly, the binica algorithm implemented in EEGLAB was used to decompose the EEG data into independent components. Dimensionality reduction was first performed using principle components analysis (30 components), which resulted in 30 independent components. Two templates were built by averaging roughly 500 manually labeled eyeblink and EOG artifact components. Then, the two components with the greatest absolute correlation with the artifact templates were selected to be removed from each BCI session (two components removed in total for each session). Finally, the data were converted to the FieldTrip data format (FieldTrip version 20180723) and noisy trials with excessive variance were removed by visual inspection^41^. Runs were broken into individual trials which included 2 s inter-trial intervals, 2 s target presentation, and a variable feedback control period. During the feedback control period, the participant was given up to 6 s to direct a cursor toward the correct target. Given the trials were all of the different lengths, we chose a consistent window of one second before the end of feedback for the analysis to ensure the consistency. Subsequently, we computed the spectral power from 1 to 50 Hz in steps of 1 Hz using a Hanning taper. Spectral power was computed for each trial and then averaged at the sensor level.

### Source analysis

We used dynamic imaging of coherent sources (DICS) to localize the underlying source of oscillatory activity^27^. By integrating the standard Colin27 MNI template, standard 10–10 EEG electrode positions, and boundary element model (BEM), the leadfield was calculated with 3898 equally distributed grid points in the gray matter. Based on the leadfield and the cross-spectral density matrix, a DICS spatial filter was then constructed to maximize the activity at the interested grid point while suppressing all the other grid points. After that, the spatial distribution of power at the source level was obtained by multiplying the spatial filter and the Fourier-transformed sensor-level data.

### Functional connectivity analysis

To assess all-to-all connectivity at the source level, we parceled the whole brain into 90 regions of interest (ROIs) based on the Automated Anatomical Labeling (AAL) template^43^. All cortical and subcortical regions within the AAL template except the cerebellum were included (Table S1). To avoid size difference biases between different ROIs, we chose the centroid within each ROI as the representative position, which is defined as the grid point with the minimum Euclidean distance to all the other grid points inside the ROI. Therefore, the frequency-specific whole-brain connectivity map has (90 × 89/2 =) 4,005 pairs of connections (90 × 90 2-D matrix). Furthermore, each ROI was assigned to one of 7 well-characterized resting-state networks^44^, derived from resting-state intrinsic connectivity analysis of 1,000 healthy participants^26^: default mode network (DMN), dorsal attention network (DAN), frontoparietal network (FPN), ventral attention network (VAN), somatomotor network (SMN), visual network (VIS) and limbic plus subcortical regions (LIM). To measure the functional connectivity, we calculated the power envelope correlation, which is suggested to be a robust and consistent method for stationary connectivity estimation^45^. The power envelope correlation method used here first orthogonalized the signals and then computed the linear correlation between these power envelopes to discount the spurious correlation caused by spatial leakage^46^.

### Statistical analysis

To assess the meditation and BCI learning interaction effect, we compared the difference of the last session and the first session between the MBSR group and the control group (MBSR_(Last-First)_ vs. Control_(Last-First)_). In the power statistics, we used the nonparametric cluster permutation test to control for multiple comparisons^47^. At the sensor level, an independent two-sample t-test (MBSR_(Last-First)_ vs. Control_(Last-First)_) was performed for each frequency and sensor, and elements with t-values exceeding the significant threshold (*P* < 0.05) were used as cluster candidates. Next, cluster candidates of sensors and frequency bins were subsequently clustered based on spatial-spectral adjacency. The spatial adjacency was defined by calculating a triangulation based on a two-dimensional projection of the sensor position. Simultaneously, the frequency bin pairs with 1 Hz difference (frequency resolution) were considered spectral adjacent. The cluster was formed by connecting the adjacent sensors and frequency bins, and at least two adjacent frequency bins and two adjacent sensors were required to construct a spatial-spectral cluster. The cluster scores were then computed as the sum of the t-values over all elements within the cluster. To get the reference distribution, we randomly shuffled the group labels 5,000 times and selected the maximum summed cluster t-statistic for each randomization. Finally, p-values were obtained by comparing the observed scores to the reference distribution (two-tailed, *p* < 0.05). The procedure was similar for the source level power statistics at a specific band, except that each cluster was formed based on spatial adjacency instead of spatial-spectral adjacency. The visualizations of power statistics at the sensor level and source level were done with Fieldtrip toolbox (https://www.fieldtriptoolbox.org/, Version: 20180805).

Network based statistics (NBS) were utilized to obtain significant functional connectivity differences^48^. Similar to the power statistics, we compared the difference of the last session and the first session between the MBSR group and the control group for each network connection using an independent two-sample t-test. Based on the pruned graphs (p < 0.005), topological clusters were formed and cluster scores were defined as the number of connections within the cluster. For the reference distribution, the cluster with the maximum number of connections was used as a test statistic. By randomizing the data across groups and recalculating the test statistic 5000 times, we generated the reference distribution, which was later used to calculate the statistics of the observed topological clusters. These statistics were conducted with the open-source NBS toolbox (http://www.nitrc.org/projects/nbs, Version: 1.2). Besides, the functional network was visualized with R Circlize package (https://cran.r-project.org/web/packages/circlize/index.html, Version: 0.4.12)^49^.

## Supplementary Information

Supplementary Information

## Data Availability

The analysis codes with sample data  are available at https://github.com/bfinl/BCI_Connectivity . Experimental data are available at 10.6084/m9.figshare.13123148.
