# Supplementary material for: Frontolimbic alpha activity tracks intentional rest BCI control improvement through mindfulness meditation
Source: Sci Rep. 2021 Mar 25;11:6818. doi: 10.1038/s41598-021-86215-0 (PMC7994299; doi:10.1038/s41598-021-86215-0)
Supplement: Supplementary file 1 — Supplementary Information [file 41598_2021_86215_MOESM1_ESM.docx]

**Supplementary Materials**

**Frontolimbic alpha activity tracks intentional rest BCI control improvement through mindfulness meditation**

Haiteng Jiang^1^, James Stieger^1,2^, Mary Jo Kreitzer^2^, Stephen Engel^2^, Bin He^1^*

1. Carnegie Mellon University, Pittsburgh, PA

2. University of Minnesota, Minneapolis, MN

**Table S1** Abbreviations for the 90 regions of interest (ROIs) used in this study

Abbreviations

Left hemisphere

Right hemisphere

Precentral gyrus PreCG.L PreCG.R

Superior frontal gyrus, dorsolateral SFGdor.L SFGdor.R

Superior frontal gyrus, orbital part ORBsup.L ORBsup.R

Middle frontal gyrus MFG.L MFG.R

Middle frontal gyrus, orbital part ORBmid.L ORBmid.R

Inferior frontal gyrus, opercular part IFGoperc.L IFGoperc.R

Inferior frontal gyrus, triangular part IFGtriang.L IFGtriang.R

Inferior frontal gyrus, orbital part ORBinf.L ORBinf.R

Rolandic operculum ROL.L ROL.R

Supplementary motor area SMA.L SMA.R

Olfactory cortex OLF.L OLF.R

Superior frontal gyrus, medial part SFGmed.L SFGmed.R

Superior frontal gyrus, medial orbital ORBsupmed.L ORBsupmed.R

Gyrus rectus REC.L REC.R

Insula INS.L INS.R

Anterior cingulate and paracingulate gyri ACG.L ACG.R

Median cingulate and paracingulate gyri DCG.L DCG.R

Posterior cingulate gyrus PCG.L PCG.R

Hippocampus HIP.L HIP.R

Parahippocampal gyrus PHG.L PHG.R

Amygdala AMYG.L AMYG.R

Calcarine fissure and surrounding cortex CAL.L CAL.R

Cuneus CUN.L CUN.R

Lingual gyrus LING.L LING.R

Superior occipital gyrus SOG.L SOG.R

Middle occipital gyrus MOG.L MOG.R

Inferior occipital gyrus IOG.L IOG.R

Fusiform gyrus FFG.L FFG.R

Postcentral gyrus PoCG.L PoCG.R

Superior parietal gyrus SPG.L SPG.R

Inferior parietal, but supramarginal and angular gyri IPL.L IPL.R

Supramarginal gyrus SMG.L SMG.R

Angular gyrus ANG.L ANG.R

Precuneus PCUN.L PCUN.R

Paracentral lobule PCL.L PCL.R

Caudate nucleus CAU.L CAU.R

Lenticular nucleus, putamen PUT.L PUT.R

Lenticular nucleus, pallidum PAL.L PAL.R

Thalamus THA.L THA.R

Heschl gyrus HES.L HES.R

Superior temporal gyrus STG.L STG.R

Temporal pole: superior temporal gyrus TPOsup.L TPOsup.R

Middle temporal gyrus MTG.L MTG.R

Temporal pole: middle temporal gyrus TPOmid.L TPOmid.R

Inferior temporal gyrus ITG.L ITG.R

Regions

**Figure S1**

**
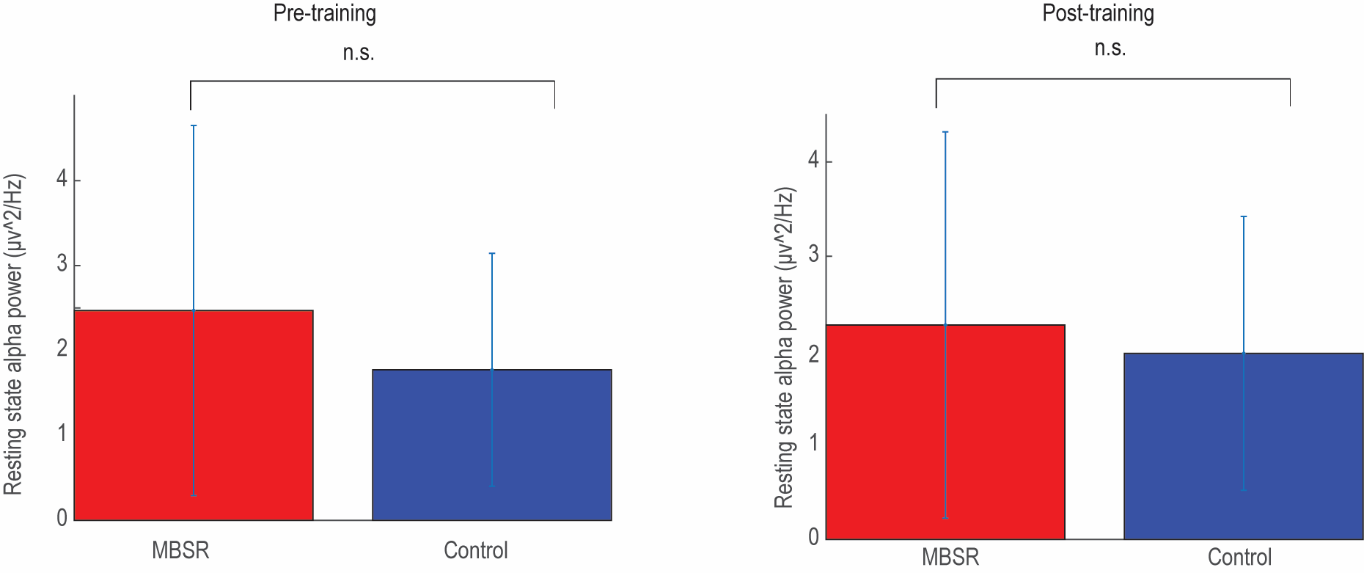
**

**Figure S1.** Pre-Training and post-training resting-state alpha power (9-15 Hz) in MBSR and Control groups. There are no significant differences between two groups in both pre-training and post-training at the *p*=0.05 level.

**Figure S2**


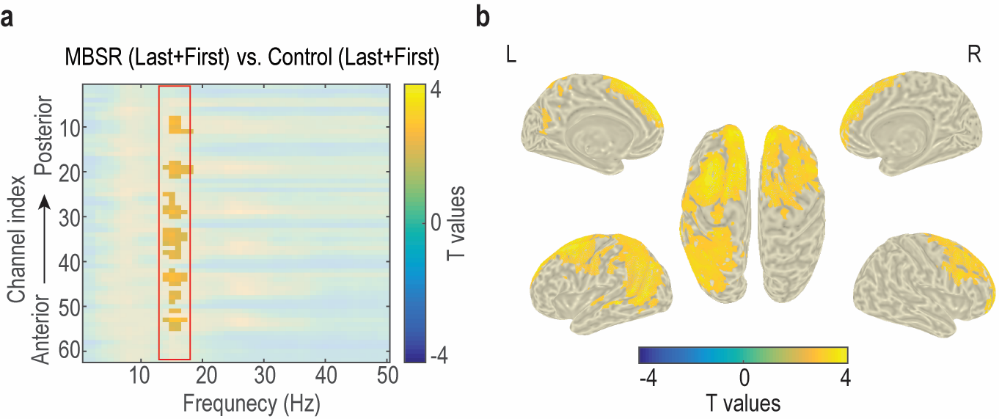


**Figure S2.** Main meditation effect in power by comparing the sum of the last session and the first session between the MBSR group and the Control group (MBSR_(Last+First)_ vs. Control_(Last+First)_). (**a**) Spectral-spatial significance map identified by cluster permutation test. Only the significant elements were highlighted. (**b**) Source localization of the 12-15 Hz oscillatory activity in the main meditation effect.

**Figure S3**


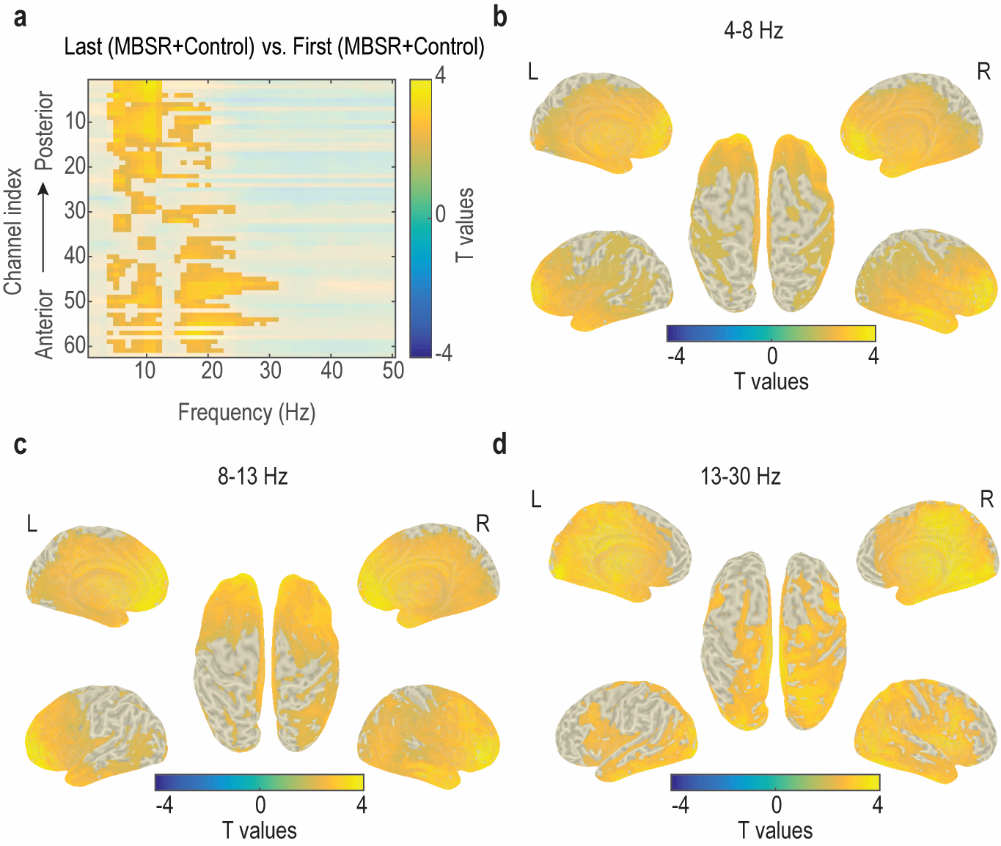


**Figure S3.** Main learning effect in power by comparing the difference between the last session and the first session in the pooled MBSR and Control group (Last_(MBSR+Control)_ vs. First_(MBSR+Control)_). (**a**) Spectral-spatial significance map identified by cluster permutation test. Only the significant elements were highlighted. (**b**) Source localization of the 4-8 Hz oscillatory activity in the learning effect. (**c**) Source localization of the 8-13 Hz oscillatory activity in the learning effect. (**d**) Source localization of the 13-30 Hz oscillatory activity in the learning effect.
